# Dental Education

**Published:** 1886-11

**Authors:** B. H. Catching


### Dental Education.

BY DR. B. H. CATCHING.

(Read before the Southern Dental Association, July 28, 1886.)

Dentistry as an art has made wonderful progress. Dentistry as a branch of medical science had advanced very slowly. The establishment of district schools for the purpose of teaching dentistry, and the creating of a separate degree, were mistakes that have, from their inception, been a drawback to our advancement in medicine. It would have been better, when we were denied the privileges of medical colleges, to have established schools of medicine and taught the whole course, conferring the degree of Doctor of Medicine, instead of going apart and establishing district schools and creating an independent degree.

It is true that we have attained to some distinction, which has been done by persistent labor at great disadvantages. This limited distinction was not acquired through those who depended alone on our schools, but on advantages obtained in schools where medicine as a whole is taught.

For fifty years we have been endeavoring to persuade the world to believe that we are specialists in medicine. That they have been so slow to believe it, is not remarkable when we consider our foundation for such assertions.

It is an unreasonable request to make on our part when we neither teach nor gradute as such, and continue to work under creatures of our own, in which medicine plays so small a part.

I do not wish to be understood as striving for medical recognition, though that would be laudable. But I do wish to be understood as desiring to place dentistry on a plane where it will command the highest respect of the people and have accorded to it its full sphere of usefulness.

It is commonly believed that the only requirement necessary for becoming a dentist, is a mechanical turn of mind, and that the only education necessary is to develop and train such a mind.

This, I am sorry to say, is not alone the opinion of a large majority of the people, but of a vast number of dentists who have been taught and practice simply the mechanical.

Our separate schools and degree have forced this conclusion on the people, and we can never change it until we change our system.

Surgery is mechanical, but it is not vulgarly accepted as a trade because it carries with it and is intimately associated with medicine. As with general surgery, so with dental surgery, if we were alike possessed of such knowledge.

If we wish to be a part of medicine and surgery, we must accept teaching necessary to qualify us for it. We must do away with separate schools and the distinct degree. We must educate and graduate from regular medical schools under one common degree.

Separate schools do not create that desire necessary for the successful prosecution of a learned profession. Their teaching is too limited for expanding the mind to nobler and higher attainments.

How can we claim to be practicing a science so high and so broad and so grand of which we hardly know the first principles?

In theoretical medicine we are taught to a very limited degree, while in chemical medicine we are not taught at all.

The establishing of boards of different kinds clearly indicates our lack in this respect. And while they may accomplish some good, yet it is not to them that we are to look for relief.

The plodder will be contented with our limited teaching, but the progressive and ambitious will look elsewhere for that knowledge not obtainable in our institutions for the proper prosecution of a branch of the healing art.

It was thought, when universities established dental departments, that every requirement had been met. When they are conducted in connection with the medical departments they are an advance toward better medical teaching, but when they are conducted apart from the medical departments they are no better than regular dental colleges, and are, in fact, nothing else.

Admitting even this to be an advance, we must not stop there. Humanity demands that we prepare ourselves more thoroughly for healing her ills and correcting her defects. Shall we longer refuse this demand? Can we afford it? I say not. We must have every advantage offered for the highest education in a calling so nearly related to divinity itself.

I will say in conclusion, do away with dental colleges; do away with university departments; do away with the degree of D.D.S. Return to the reputable medical colleges, enter and come forth as doctors of medicine, and then enter a dental infirmary in which the highest type oı practical dentistry is taught, and then we will stand fully equipped to practice dentistry as a specialty of medicine.

I do not wish to convey the idea that we should accept the teaching of every medical college; far from it. There are too many of them nothing but catch-penny establishments, caring nothing for the manner of their teaching, and less for the qualification of their graduates. While we are not here to read lessons to medical colleges, yet we can appropriately urge them to establish chairs of dental surgery and teach it as applied to medicine. It would not only be a vast benefit to the physicians so taught, and a blessing to the people, but it would cause a higher appreciation of educated dentistry, and redound to our good as well as to their glory.

